# GC-MS Analysis and Preliminary Antimicrobial Activity of *Albizia adianthifolia* (Schumach) and *Pterocarpus angolensis* (DC)

**DOI:** 10.3390/medicines3010003

**Published:** 2016-01-29

**Authors:** Mustapha N. Abubakar, Runner R. T. Majinda

**Affiliations:** Chemistry Department, University of Botswana, P/Bag 0022, Gaborone, Botswana; mngaskyii@yahoo.com

**Keywords:** GC-MS, NMR, antimicrobial activity, *A. adianthifolia*, *P. angolensis*

## Abstract

The non-polar components of two leguminoceae species *Albizia adianthifolia* (Schumach), and *Pterocarpus angolensis* (DC) were investigated. GC-MS analysis of the crude *n*-hexane and chloroform extracts together with several chromatographic separation techniques led to the identification and characterization (using NMR) of sixteen known compounds from the heartwood and stem bark of *Albizia adianthifolia* and *Pterocarpus angolensis* respectively. These constituents include, *n*-hexadecanoic acid (palmitic acid) **1**, oleic acid **2**, chondrillasterol **3**, stigmasterol **4**, 24S 5α-stigmast-7-en-3β-ol **5**, 9,12-octadecadienoic acid (*Z*,*Z*)-, methyl ester **6**, *trans-*13-octadecanoic acid, methyl ester **7**, tetradecanoic acid **8**, hexadecanoic acid, methyl ester **9**, octadecanoic acid **10**, tetratriacontane **11**, 7-dehydrodiosgenin **12**, lupeol **13**, stigmasta-3,5-diene-7-one **14**, friedelan-3-one (friedelin) **15**, and 1-octacosanol **16**. Using agar over lay method, the preliminary antimicrobial assay for the extracts was carried out against bacterial (*E. coli*, *P. aeruginosa*, *B. subtilis*, *S. aueus*) and a fungus/yeast (*C. albicans*) strains. The *n*-hexane and chloroform extracts of *A. adianthifolia* showed the best activity against *E. coli* with minimum inhibition quantity (MIQ) of 1 µg each while the remaining exhibited moderate-to-weak activity against the test microorganisms.

## 1. Introduction

The non-polar extracts of aromatic medicinal plant species are mainly composed of essential oils (EOs) and other complex mixture of compounds, mainly monoterpenes, sesqiuterpenes, long chain aliphatics, and their oxygenated derivatives (alcohols, aldehydes, esters, ethers, ketones, phenols, and oxides). These constituents may be obtained from different plant materials such as flowers, bark, wood, twigs, leaves, and roots [1].

The genus Albizia is comprised of about 150 species widely distributed in tropics, with great diversity in Africa and Central South America [2]. *Albizia adianthifolia* (Schumach), (Family: Fabaceae, Mimosoideae), is a large deciduous tree, trivially known as flat crown Albizia, that grows and habitats well in both dry woodland and sandy soils. It is valued as a shade tree in cocoa and coffee plantations in Sierra Leone as well as for soil conservation. Amongst other economic important uses of this species is its utilization in light constructions (e.g., rafters, posts) and carvings (e.g., spoons, clubs). Extensive literature survey revealed that various parts of *A. adianthifolia* have been used for a number of folkloric applications to treat such diseases such as skin and respiratory tract infections, headaches, sinusitis, and malaria, as well as use as analgesic, purgative, anti-inflammatory, and as a psychotic principle [3,4].

*Pterocarpus* genus is comprised of 100 species amongst which is included *Pterocarpus angolensis* (DC), (Family: Fabaceae, Papillionoideae)*. P. angolensis* is mainly found in the tropical regions of Africa as well as the Neotropical regions and Indomalaya [5]. It is a deciduous large tree, 18–19 m tall with a dark brown bark and a high wide-crowned canopy of shiny compound leaves. The leaves, which are alternate, deep green and imparipinnate, appear at the time of flowering or shortly afterwards. The pod has a diameter of about 2–3 cm, is surrounded by a circular wing which is 8–12 cm in diameter, reminiscent of a brown fried egg, and contains a single seed. This characteristic brown papery and spiky seed pod stays on long after the leaves have fallen from the tree. *P. angolensis* grows well on deep sandy soils or well drained rocky slopes and on open woodland areas. Owing to its excellent general properties the timber from *P. angolensis* has many applications in the building and furniture industries. *P. angolensis* is a woody tree that is revered because of its great natural durability [6]. The sticky deep red/maroon colored sap is used as dye for fabric. In folk medicine, *P. angolensis* is mainly used for gastro-intestinal, urino-genital, fertility problems, respiratory conditions, and skin afflictions [7]. The traditional use of extracts from *A. adianthifolia* and *P. angolensis* seem to suggest, among others, efficacy against free radical induced and microbial infections.

There are literature reports on the composition of non-polar constituents from both Albizia and Pterocarpus generra. For example, the GC-MS analysis of the ethanolic and *n*-hexane extracts of the stem bark of *A. chevalieri* [8] and the ethanolic extracts of the stem bark and heartwood of *P. marsupium* along with their identified (antimicrobial, antioxidant, preservative, lubricant, flavor *etc.*) activities were reported [9]. To the best of our knowledge, no study concerning the non-polar extracts of the heartwood and stem bark of either *A. adianthifolia* or *P. angolensis* has been reported.

Due to the diversity of folkloric uses of *A. adianthifolia* and *P. angolensis* against a number of diseases, we considered it worthwhile to determine the non-polar constituents of these two plant species. This was done by GC-MS analysis of the non-polar *n*-hexane and chloroform extracts and identification of the components was achieved by comparison of the acquired spectra with the existing NIST library. We were able to isolate other constituents using conventional column chromatography whose structures were elucidated using NMR spectroscopy. The presence of some of these constituents was later confirmed by GC-MS analysis. The GC-MS analysis of the non-polar constituents, not surprisingly, revealed that some components were in fairly high concentrations (major components) while other were only present in trace amounts [10].

## 2. Materials and Methods

Analytical reagents (AR) and general purpose reagents (GPR) solvents were used. The GPR solvents were distilled using simple distillation before use.

### 2.1. Collection of Plant Materials

The heartwood of *A. adianthifolia* was collected from Sokoto, north-western Nigeria in January, 2011. The plant species was identified by Mal. Auwal Muhammad, Botany Unit, Biological Sciences, Usmanu Danfodiyo University Sokoto, Nigeria and a voucher specimen, UDUH/ANS/0029, was deposited in the University Herbarium. The stem bark of *P. angolensis* was collected from Kasane in the northern region of Botswana in June 2012 and was identified at the Botany unit of Biological Sciences, University of Botswana where a voucher specimen was also deposited. The samples were air dried under shade, pulverized into course powder using Thomas Wiley Model 4 and stored in air tight containers until use.

### 2.2. Extraction Method

The heartwood of *A. adianthifolia* (657.51 g) was successively and exhaustively extracted with *n*-hexane, chloroform, methanol, and 10% methanol (aq). The *n*-hexane and chloroform extracts, upon concentration under reduced pressure using a rotavapor (Buchi, Flawil, Switzerland) (<40 °C), yielded respectively orange-yellow (0.95 g) and yellowish-green (9.16 g) crude extracts. Following a similar extraction protocol the stem bark of *P. angolensis* (2.21 kg) yielded *n*-hexane (yellow paste; 51.45 g), and chloroform (reddish-brown paste; 11.6 g) crude extracts.

A small portion of the *n*-hexane crude extract from each species was dissolved in chloroform and subjected to GC-MS analysis. The main crude chloroform extract from each of the species was separately adsorbed on coarse silica gel (0.2–0.5 mm) ratio (1:1), and allowed to dry before packing or loading onto a column with fine silica gel 60 (0.040–0.063 mm). The extracts were separately eluted under vacuum liquid chromatography (VLC) using *n*-hexane-chloroform-methanol in increasing (10%) polarity until 100% methanol.

The crude chloroform extract of the heartwood *of A. adiantifolia* yielded 52 fractions (50 mL each). These fractions were pooled based on the analytical TLC profiling (using acetone/*n*-hexane, 3:7) to give fractions 1–9 which were encoded ‘’**A**’’. Fraction ‘’**A**’’ was then subjected to GC-MS analysis. Using the same procedure, the crude chloroform extract of the stem bark of *P. angolensis* yielded 79 fractions. These were combined to give sub-fractions **A**–H. Sub-fraction F was adsorbed on coarse silica gel and subjected to column chromatography using different solvent systems (*n*-hexane/chloroform/acetone) in increasing (10%) polarity to yield 19 sub-fractions. These were combined which further yielded eight sub-fractions encoded F1–F8. From sub-fraction F3, a white crystalline solid (15.5 mg) was obtained which was identified by NMR spectroscopy as stigmasterol. The presence of stimasterol in the crude chloroform extract was confirmed by GC-MS analysis.

### 2.3. Procedure for GC-MS Analysis

The *n-*hexane extracts obtained from the heartwood and stem bark of *A. adianthifolia* and *P. angolensis* respectively were analyzed separately by GC-MS using a HP-5MS capillary column (30 m × 250 μm, i.d., 0.25 μm film thickness) in an Agilent 6890N gas chromatograph (Agilent Technologies, Palo Alto, CA, USA) coupled to a water GCT Premier mass spectrometer (Waters Corporation, Milford, MA, USA). The carrier gas was helium with a constant flow rate of 3 mL/min. The oven temperature was initially kept at 100 °C for 4 min then ramped at 10 °C/min to 240 °C. The temperature was gradually increased from 8 °C/min to 300 °C and held isothermally for 10 min. An amount of 1.0 μL of the sample (100 ppm in chloroform) solutions was injected in the splitless mode. Mass spectra were obtained by EI at 70 eV over the scan range *m*/*z* 50−800. The compounds were identified by comparison of their mass spectra with those of the NIST 05 L mass spectral library. The spectral match factor limit was set at 700 and any components with match factor less than 700 were not considered [11].

### 2.4. Procedure for Antimicrobial Activity

The antimicrobial activities of the extracts were evaluated using the modified agar overlay method [12,13,14]. The nutrient broth was prepared by dissolving (13 g/L) in distilled water and heating on a hotplate equipped with magnetic stirrer until a homogenous mixture was obtained. Then 50 mL of the nutrient broth was transferred into 250 mL (×5 for each organism) Erlenmeyer flasks and stoppered with cotton wool and aluminum foil. The nutrient agar was prepared by dissolving (28 g/L) in distilled water in a similar manner as the nutrient broth. The two (nutrient broth and nutrient agar) were separately autoclaved for 15 min at 121 °C. The nutrient broth was cooled in a Biohazard cabinet while the nutrient agar was kept in an oven set at 45 °C until ready to use.

Suspensions (10 mL) of the reconstituted pathogens were separately introduced into labeled 250 mL (×5) Erlenmeyer flasks containing 100 mL warm nutrient agar. Using sterile graduated pipettes, the pathogens were administered and spread as evenly as possible onto the pre-coated silica gel TLC plates already loaded with the different compounds in various loadings *i.e.*, 100, 50, 10, 5, 1 (μg). The nutrient agar containing the pathogens administered was allowed to solidify before being incubated for 24 h at 37 °C and 28 °C for the bacterial and fungal strains respectively. The zones of inhibitions (in “mm” after 24 h) were measured after staining of the plates with methylthiazolyltetrazolium chloride (MTT—2 mg/mL). Chloramphenicol and miconazole were used as standards for the bacterial and fungal strains respectively. The entire microbial assay was conducted under strict aseptic conditions.

## 3. Results and Discussions

### 3.1. GC-MS Analysis

GC-MS analysis of the volatile chemical compositions of *A. adianthifolia* (heartwood) and *P. angolensis* (stem bark) from the *n-*hexane and chloroform extracts were highly complex containing glycosides, ketone, saturated and unsaturated fatty acids, alcohols, and sterols (Table 1 and Table 2). These constituents were presented according to the extracts from which they were identified in the next sections of this report.

#### 3.1.1. GC-MS Analysis of *A. adianthifolia*

**Figure 1:**
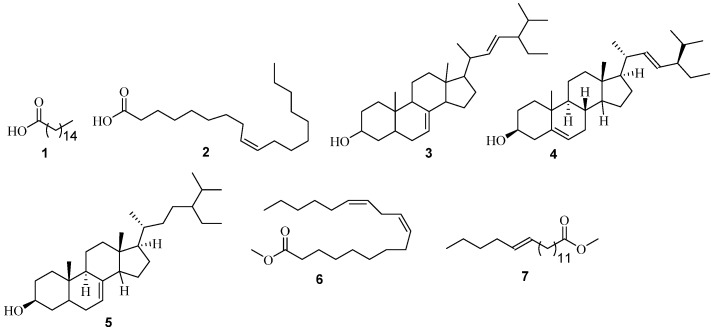
Structures of non-polar constituents identified from *A. adianthifolia* using GC-MS analysis.

Seven constituents were predominantly found in the heartwood of *A. adianthifolia* (Figure 1, see also Figures S1 and S2). The peak areas (or percentage compositions of the metabolites shown in the brackets) are relative to other constituents within the crude extracts and whose match factors were greater than 700. Five of these were from the *n*-hexane extract. They include *n*-hexadecanoic acid **1** (34.85%), stigmasterol **4** (28.64%), oleic acid **2** (6.28%), 24S 5α-stigmast-7-en-3β-ol **5** (4.37%), chondrillasterol **3** (18.23%). While the remaining two—*i.e.*, 9,12-octadecadienoic acid (Z,Z)-, methyl ester **6** (17.58%) and *trans*-13-octadecenoic acid, methyl ester **7** (37.23%)—were from the chloroform extract (Table 1). Most of these constituents have been found to show interesting biological activity against certain illnesses and/or pathogens. For instance, the anti-inflammatory [15], antioxidant, hypocholesterolemic [16], antibacterial [17], activities reported for *n*-hexadecanoic acid **1**, may suggest the rationale for the traditional use of the species.

**Table 1:** Phyto-constituents identified from heartwood of *A. adianthifolia* by GC-MS analysis.

Species	Compd. Name/No	Mol. Formula	Mol. Wt.	NIST Match Factor: Forward, Reverse	RT (min)	Peak Area (%)	Reported Bioactivity
*A. adianthifolia* (*n*-hexane extract)	*n*-hexadecanoic acid 1	C_16_H_32_O_2_	256	858, 880	16.65	34.85	Anti-inflammatory [15], Antioxidant, hypocholesterolemic nematicide, pesticide, anti androgenic flavor, hemolytic, 5-Alpha reductase inhibitor [16], potent mosquito larvicide [17].
	oleic acid 2	C_18_H_34_O_2_	282	754, 892	18.27	6.28	Antibacterial [18].
	chondrillasterol 3	C_29_H_48_O	412	805, 935	29.29	18.23	Cytotoxicity [19].
	stigmasterol 4	C_29_H_48_O	412	818, 830	29.30	28.64	Thyroid inhibitory, antiperoxidative and hypoglycemic effects [20].
	24S, 5α stigmast-7-en-3β-ol 5	C_29_H_50_O	414	809, 930	30.02	4.37	Antimutagenic [21].
Total = 92.37							
*A. adianthifolia* (chloroform extract)	9,12-octadecadienoic acid (Z,Z)-, methyl ester 6	C_19_H_34_O_2_	294	750, 895	17.76	17.58	Anti-cancer [22].
	*trans*-13-octadecanoic acid, methyl ester 7	C_19_H_36_O_2_	296	857, 907	17.81	37.23	Anti-inflammatory, antiandrogenic, cancer preventive, dermatitigenic, irritant, antileukotriene—D4, hypocholesterolemic, 5-alpha reductase inhibitor, anemiagenic, insectifuge, flavor [23].
Total = 54.81							

#### 3.1.2. GC-MS Analysis of *P. angolensis*

**Figure 2:**
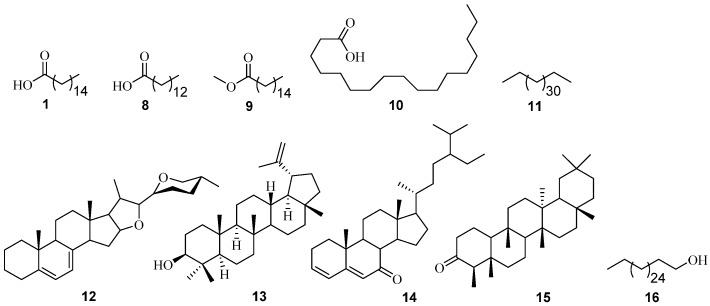
Structures of non-polar constituents identified from *P. angolensis* using GC-MS analysis.

Ten volatile phytoconstituents were found to be the most abundant in the *n-*hexane extract of the stem bark of *P. angolensis* (Figure 2). These constituents and their calculated percentage peak area compositions include tetratriacontane **11** (31.67%), *n*-hexadecanoic acid **1** (10.29%), 7-dehydrodiosgenin **12** (9.58%), stigmasta-3,5-dien-7-one **14** (7.13%), lupeol **13** (6.54%), octadecanoic acid **10** (5.89%), friedelan-3-one **15** (2.56%), hexadecanoic acid, methyl ester **9** (1.84%), and tetradecanoic acid **8** (1.84%). While from the chloroform extract, 1-octacosanol **16** (9.87%), was isolated after several chromatographic separation techniques, elucidated using NMR spectroscopic data and confirmed/identified via GC-MS analysis (Table 1 and Table 2, see also Figures S3 and S4).

**Table 2:** Phytocomponents identified from the stem bark of *P. angolensis* by GC-MS analysis.

Species	Compd. Name/No.	Mol. Formula	Mol. Wt.	NIST Match Factor: Forward, Reverse	RT (min)	Peak Area (%)	Reported Bioactivity
*P. angolensis* (*n*-hexane extract)	tetradecanoic acid **8**	C_14_H_28_O_2_	228	835, 902	14.52	1.84	Larvicidal and repellent activity [24].
	hexadecanoic acid, methyl ester **9**	C_17_H_34_O_2_	270	879, 903	16.15	1.84	Antibacterial and antifungal [25]
	*n*-hexadecanoic acid **1**	C_16_H_32_O_2_	256	864, 879	16.70	10.29	See above
	octadecanoic acid **10**	C_18_H_36_O_2_	284	780, 825	18.48	5.89	Antimicrobial activity [17].
	tetratriacontane **11**	C_34_H_70_	478	759, 874	24.79	31.67	Antibacterial and antifungal [26]
	7-dehydrodiosgenin **12**	C_27_H_40_O_3_	412	721, 736	26.59	9.58	Antibacterial, antifungal,, antioxidant, cytotoxic [27,28]
	lupeol **13**	C_30_H_50_O	426	832, 897	30.48	6.54	Anti-inflammatory activity [29], Anti-cancer [30].
	stigmasta-3,5-dien-7-one **14**	C_29_H_46_O	410	817, 864	30.66	7.13	Free radical scavenging? Anti-diabetic, anticancer? [31,32]
	friedelan-3-one **15**	C_30_H_50_O	426	882, 903	32.91	2.56	Antibacterial, antifungal, anti-inflammatory, analgesic, antipyretic, antihypertensive [33,34]
Total = 77.34							
*P. angolensis* (chloroform extract)	* 1-octacosanol **16**	C_28_H_58_O	410	837, 918 813, 891	26.44 28.61	37.73 62.27	Antioxidant [35].
Total = 100							

* Same compound but appeared at two different RT (min) showing two distinct peaks on the spectrum and with different % compositions.

The compound *n*-hexadecanoic acid **1** was found in both extracts from the different species. It may be inferred that both species have similarities in terms of their traditional applications and effectiveness against such illnesses as skin diseases and respiratory conditions and as anti-inflammatories and analgesics may be due to the presence of *n*-hexadecanoic acid **1**.

### 3.2. Structure Elucidation of Compound **16**

The proton-decoupled ^13^C-NMR showed an envelope of carbons resonating at **δ**_C_ 29.7–29.4 in addition to six other carbon signals which from the DEPT 135 spectrum gave one methyl at δ_C_ 14.1, the remaining five were methylene carbons which resonated between δ_C_ 32.9–22.7. However, except for the methyl carbon at δ_C_ 14.1, and the carbon that resonated at δ_C_ 63.1 which is characteristic of an oxygenated aliphatic methylene carbon, the rest were typical sp^3^ aliphatic methylene carbons. From the ^1^H-NMR, a two proton broad singlet at δ_H_ 3.69 (characteristic of an oxygenated aliphatic environment), another triplet of three protons appearing up field δ_H_ 0.93 (typical of a terminal aliphatic methyl) were observed. Three apparent singlets were observed at δ_H_ 2.22, δ_H_ 1.63, and δ_H_ 1.30 due possibly to insufficient resolution on a 300 MHz NMR. These apparent singlet signals (which should have been quintets or at worst multiplets) were characteristic of sp^3^ aliphatic methylene protons (Table 3, see also Figures S5 and S6). These data are consistent with a long aliphatic chain attached to a hydroxyl group. Further confirmation was obtained from the GC-MS library data match. Thus, compound **16** was identified as 1-octacosanol.

**Table 3:** ^1^H (300 MHz) and ^13^C (75 MHz) NMR spectra of 16 in CDCl_3_.

Position	δ_H_(ppm)	δ_C_ (ppm)
1	3.69, 2H, *br s* *	63.1
2	2.22, 2H, *s* *	32.9
3	1.63, 2H, *s* *	32.0
4–27	1.35–1.30, 49H, *s*	29.7–22.7
28	0.93, 3H, *t,* (6.6)	14.1

* = apparent singlet.

### 3.3. Result for the Preliminary Antimicrobial Assay

The bioactivity reported (from other species) for these metabolites identified from the crude extracts of *A. adianthifolia* and *P. angolensis* against a variety of diseases (Table 1 and Table 2), necessitated the preliminary antimicrobial assay. The preliminary antimicrobial assay of the extracts showed different responses to the test organisms with best activity observed for both *n*-hexane and chloroform extracts of *A. adianthifolia* against *E. coli* with MIQ of 1 μg (Table 4). The *P. angolensis* stem bark extracts were observed to show poor-to-no activity against the test pathogens except for the weak response of the chloroform extract against *B. subtilis* with MIQ of 50 μg. The preliminary antimicrobial potency of these species is generally characterized by weak to poor activity as none of the extracts demonstrated activity comparable to the standards used (*i.e.*, chloramphenicol and miconalzole).

**Table 4:** Preliminary antimicrobial activity of the different extracts.

Species	Part	Extract	Microbial Strains and MIQ (μg)				
			Gram+ve Bacteria		Gram−ve Bacteria		Fungus
			*E. coli*	*P. aeruginosa*	*B. subtilis*	*S. aureus*	*C. albicans*
*A. adianthifolia*	heartwood	*n*-hexane	1	50	50	50	>100
*A. adianthifolia*	heartwood	Chloroform	1	50	50	50	>100
*P. angolensis*	stem bark	*n*-hexane	>100	>100	100	>100	100
*P. angolensis*	stem bark	Chloroform	>100	>100	50	>100	100
Chloramphenicol			0.50	10	0.25	0.50	N.A
Miconazole			N.A	N.A	N.A	N.A	0.25

Not active at a loading >100 μg; N.A = not applicable. MIQ = minimum inhibition quantities.

## 4. Conclusions

The GC-MS analysis showed the presence of sixteen phytochemical constituents from the non-polar and medium polar extracts of the heartwood and stem bark of the *A. adianthifolia* and *P.* *angolensis* respectively. Although the preliminary antimicrobial assay did not convincingly corroborate with the acclaimed folkloric uses with only moderate to weak activity exhibited by the pathogens tested, it could be considered that the efficacy of these species in certain illnesses could be associated with the presence of similar phyto-constituents (*i.e.*, *n*-hexadecanoic acid 1). Detailed phytochemical investigation should be carried out on the polar extracts to identify other constituents that may have led to the popular use of these species in folklore medicine.

## Supplementary Materials

The following are available online at www.mdpi.com/2305-6320/1/3/3/s1, Figure S1: GC-MS chromatogram of the *n*-hexane (heartwood) extract of *A. adianthifolia*, Figure S2: GC-MS chromatogram of Sub-fraction “A” of the chloroform (heartwood) extract of *A. adianthifolia*, Figure S3: GC-MS chromatogram of the *n*-hexane (stem bark) extract of *P. angolensis*, Figure S4: GC-MS chromatogram of the sub-fraction F3 of the chloroform (stem bark) extract of *P. angolensis*, Figure S5: **^1^**H (300 MHz) NMR spectra of 1-octacosanol **16** in CDCl_3_, Figure S6: ^13^C (75 MHz) NMR spectra of 1-octacosanol **16** in CDCl_3_.

## References

[1] Delamare A.P.L., Moschen-Pistorello I.T., Artico L., Atti-Serafini L., Echeverrigaray S. (2007). Antibacterial activity of the essential oils of *Salvia officinalis* L. and *Salvia triloba* L. cultivated in South Brazil. Food Chem..

[2] Abdel-Kader M., Hoch J., Berger J.M., Evans R., Miller J.S., Wisse J.H., Mamber S.W., Dalton J.M., Kingston D.G.I. (2001). Two bioactive saponins from *Albizia subdimidiata* from the Suriname rainforest. J. Nat. Prod..

[3] Lawal I.O., Uzokwe N.E., Igboanugo A.B.I., Adio A.F., Awosan E.A., Nwogwugwu J.O., Faloye B., Olatunji B.P., Adesoga A.A. (2010). Ethno-medicinal information on collation and identification of some medicinal plants in research institutes of South-west Nigeria. Afr. J. Pharm. Pharmacol..

[4] Tamokou J., Mpetga D.J.S., Lunga P.K., Tene M., Tane P., Kuiate J.R. (2012). Antioxidant and antimicrobial activities of ethyl acetate extract, fractions and compounds from stem bark of *Albizia adianthifolia* (*Mimosoideae*). BMC Complement. Altern. Med..

[5] Rojo J.P. (1972). Pterocarpus (Leguminoseae-Papillinaceae) Revised for the World.

[6] King F.E., King T.J., Warwick A.J. (1952). The chemistry of extractives from hardwoods. Part VI. Constituents of muninga, the heartwood of *Pterocarpus Angolensis*, A. : 6: 4′-dihydroxy-5 : 7-dimethoxyisojavone (muningin). J. Chem. Soc..

[7] Saslis-Lagoudakis C.H., Klitgaard B.B., Forest F., Francis L., Savolainen V., Williamson E.M., Hawkins J.A. (2011). The use of phylogeny to interpret cross-cultural patterns in plant use and guide medicinal plant discovery: An example from *Pterocarpus* (Leguminosae). PloS One.

[8] Ama I.U., David C.O., UcheOrji O., Maduabuchi A.P., Chukwu C., Obasi J.N. (2015). GC-MS analysis, acute toxicity and oxidative stress potentials (effects) of *Albizia chevalieri* extract on juvenile African catfish (*Clarias gariepinus*). Middle East J. Sci. Res..

[9] Maruthupandian A., Mohan V.R. (2011). GC-MS analysis of some bioactive constituents of *Pterocarpus marsupium* Roxb. Int. J. Chem. Tech. Res..

[10] Bakkali F., Averbeck S., Averbeck D., Idaomar M. (2008). Biological effects of essential oils—A review. Food Chem. Toxicol..

[11] Watson J.T., Sparkman O.D. (2007). Introduction to Mass Spectrometry: Instrumentation, Applications, and Strategies for Data Interpretation.

[12] Saxena G., Farmer S., Towers G.H.N., Hancock R.E.W. (1995). Use of specific dyes in the detection of antimicrobial compounds from crude plant extracts using a thin layer chromatography agar overlay technique. Phytochem. Anal..

[13] Rahalison M., Hamburger M., Hostettmann K. (1991). A bioautographic agar overlay method for the detection of antifungal compounds from higher plants. Phytochem. Anal..

[14] Cos P., Vlietinck A.J., Berghe D.V., Maes L. (2006). Anti-infective potential of natural products: How to develop a stronger *in vitro* “proof-of-concept”. J. Ethnopharmacol..

[15] Aparna V., Dileep K.V., Mandal P.K., Karthe P., Sadasivan C., Haridas M. (2012). Anti-inflammatory property of *n*-hexadecanoic acid: Structural evidence and kinetic assessment. Chem. Biol. Drug Des..

[16] Kumar P.P., Kumaravel S., Lalitha C. (2010). Screening of antioxidant activity, total phenolics and GC-MS study of *Vitex negundo*. Afr. J. Biochem. Res..

[17] Rahuman A.A., Gopalakrishnan G., Ghouse B.S., Arumugam S., Himalayan B. (2000). Effect of *Feronia limonia* on mosquito larvae. Fitoterapia.

[18] Awa E.P., Ibrahim S., Ameh D.A. (2012). GC/MS analysis and antimicrobial activity of diethyl ether fraction of methanolic extract from the stem bark of *Annona senegalensis* Pers. Int. J. Pharm. Sci. Res..

[19] Chen J.J., Duh C.Y., Chen I.S. (2005). Cytotoxic chromenes from *Myriactis humilis*. Planta Med..

[20] Panda S., Jafri M., Kar A., Meheta B.K. (2009). Thyroid inhibitory, antiperoxidative and hypoglycemic effects of stigmasterol isolated from *Butea monosperma*. Fitoterapia.

[21] Guevara A.P., Amor E., Russell G. (1996). Antimutagens from *Plumeria acuminata* ait. Mutat. Res. Environ. Mutagen. Relat. Subj..

[22] Yu F.R., Lian X.Z., Guo H.Y., McGuire P.M., Li R.D., Wang R., Yu F.H. (2005). Isolation and characterization of methyl esters and derivatives from *Euphorbia kansui* (*Euphorbiaceae*) and their inhibitory effects on the human SGC-7901 cells. J. Pharm. Pharm. Sci..

[23] Krishnamoorthy K., Subramaniam P. (2014). Phytochemical profiling of leaf, stem, and tuber parts of *Solena amplexicaulis* (Lam.) Gandhi Using GC-MS. Int. Sch. Res. Not..

[24] Sivakumar R., Jebanesan A., Govindarajan M., Rajasekar P. (2011). Larvicidal and repellent activity of tetradecanoic acid against *Aedes aegypti* (Linn.) and *Culex quinquefasciatus* (Say.) (Diptera: Culicidae). Asian Pac. J. Trop. Med..

[25] Chandrasekaran M., Senthilkumar A., Venkatesalu V. (2011). Antibacterial and antifungal efficacy of fatty acid methyl esters from leaves of *Sesuvium portulacastrum* L.. Eur. Rev. Med. Pharmcol. Sci..

[26] Mahmood A., Ahmed R., Kosar S. (2009). Phytochemical screening and biological activities of the oil components of *Prunus domestica* Linn. J. Saudi Chem. Soc..

[27] Reddy K.S., Shekhami M.S., Berry D.E., Lynn D.G., Hecht S.M. (1984). Afrontoside a new cytotoxic principle from *Dracaena afromontana*. J. Chem. Soc. Perkin Trans..

[28] Karabay-Yavasoglu N.U., Sukatar A., Ozdemir G., Horzum Z. (2007). Antimicrobial activity of volatile components and various extracts of the red algae *Jania rubens*. Phytother. Res..

[29] Geetha T., Varalakshmi P. (2001). Anti-inflammatory activity of lupeol and lupeol linoleate in rats. J. Ethnopharmacol..

[30] Saleem M. (2009). Lupeol, a novel anti-inflammatory and anti-cancer dietary triterpene. Cancer Lett..

[31] Balogun O.S., Oladosu I.A., Akiinnusi A., Zhiqiang L. (2013). Fatty acid composition α-glucosidae inhibitory potential and cytotoxicity activity of *Oncoba spinosa* Forssk. Elix. Appl. Chem..

[32] Delazar A., Nazifi E., Movafeghi A., Nazemiyey H., Hemmati S., Nahar L., Sarker S.D. (2010). Analyses of phytosterols and free radical scavengers in the bulbs of *Ornithogalum cuspidatum* Bertol. Bol. Latinoam. Caribe Plant. Med. Aromat..

[33] Antonisamy P., Duraipandiyan V., Ignacimuthu S. (2011). Anti-inflammatory, analgesic and antipyretic effects of friedelin isolated from *Azima tetracantha* Lam. in mouse and rat models. J. Pharm. Pharmacol..

[34] Ghosh P., Chakraborty P., Basak G. (2011). Antibacterial, antifungal and phytotoxic screening of some prepared pyrazine derivatives in comparison to their respective α-diketo precursors. Int. J. Pharm. Sci. Res..

[35] Firdous S., Khan K., Zikr-Ur-Rehman S., Ali Z., Soomro S., Ahmad V.U., Rasheed M., Mesaik M.A., Faizi S. (2014). Isolation of phytochemicals from *Cordia rothii* (Boraginaceae) and evaluation of their immunomodulatory properties. Rec. Nat. Prod..

