# Supplementary material for: GC-MS Analysis and Preliminary Antimicrobial Activity of Albizia adianthifolia (Schumach) and Pterocarpus angolensis (DC)
Source: Medicines (Basel). 2016 Jan 29;3(1):3. doi: 10.3390/medicines3010003 (PMC5456228; doi:10.3390/medicines3010003)
Supplement: Supplementary file 1 [file medicines-03-00003-s001.pdf]

Supplementary Materials: GC-MS Analysis and Preliminary Antimicrobial Activity of *Albizia adianthifolia* (Schumach) and *Pterocarpus angolensis* (DC)

Mustapha N. Abubakar and Runner R. T. Majinda

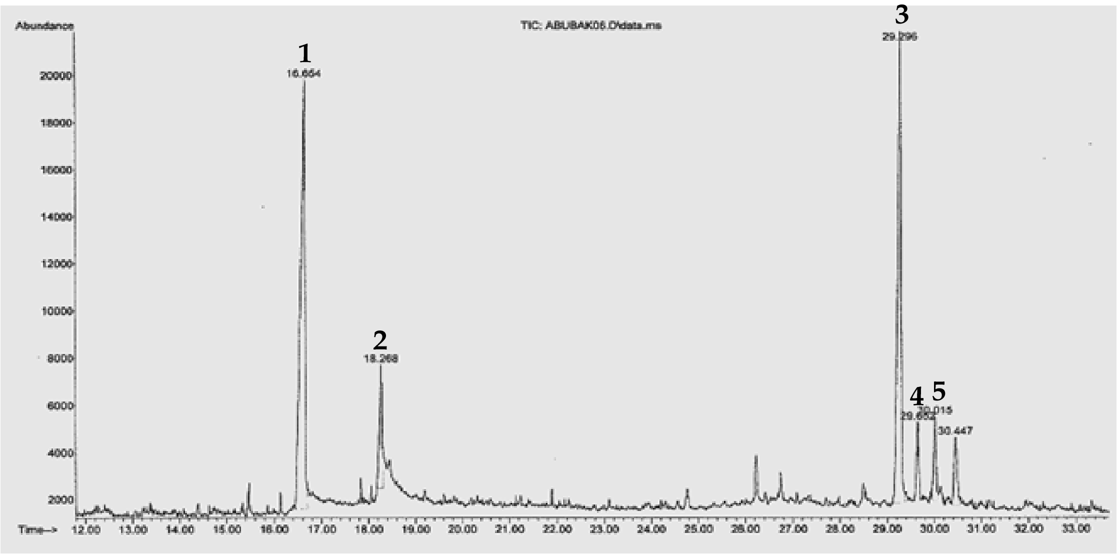

Figure S1. GC-MS chromatogram of the *n*-hexane (heartwood) extract of *A. adianthifolia.*

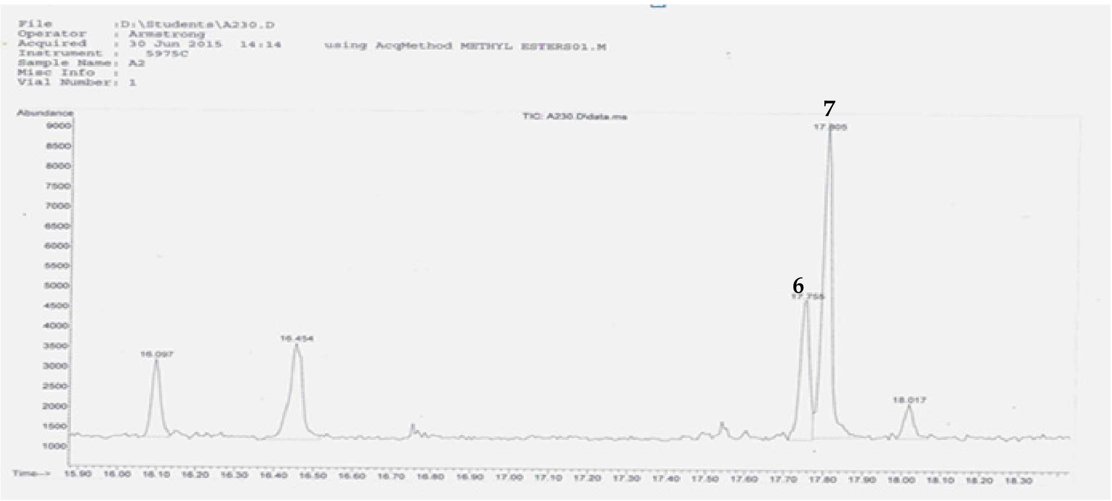

Figure S2. GC-MS chromatogram of Sub-fraction "A" of the chloroform (heartwood) extract of *A. adianthifolia*.

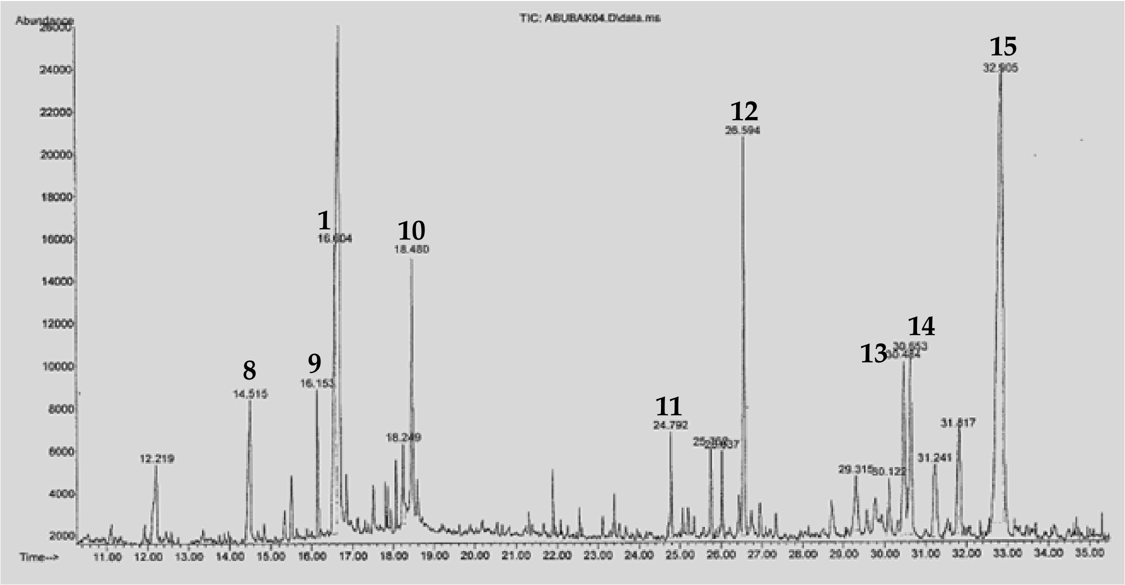

Figure S3. GC-MS chromatogram of the *n*-hexane (stem bark) extract of *P. angolensis*.

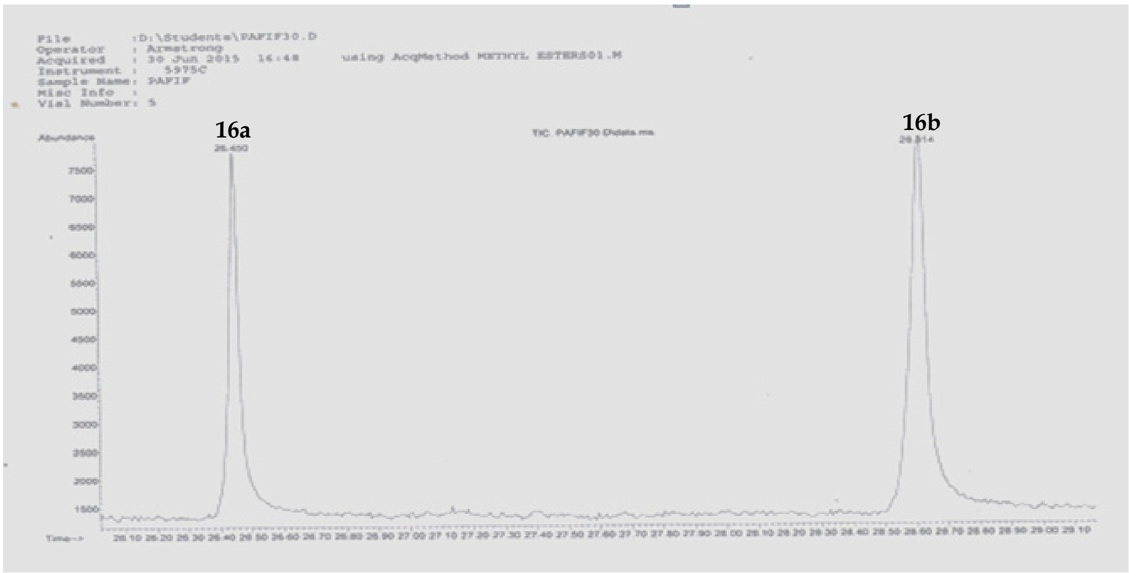

Figure S4. GC-MS chromatogram of the sub-fraction F3 of the chloroform (stem bark) extract of *P. angolensis.*

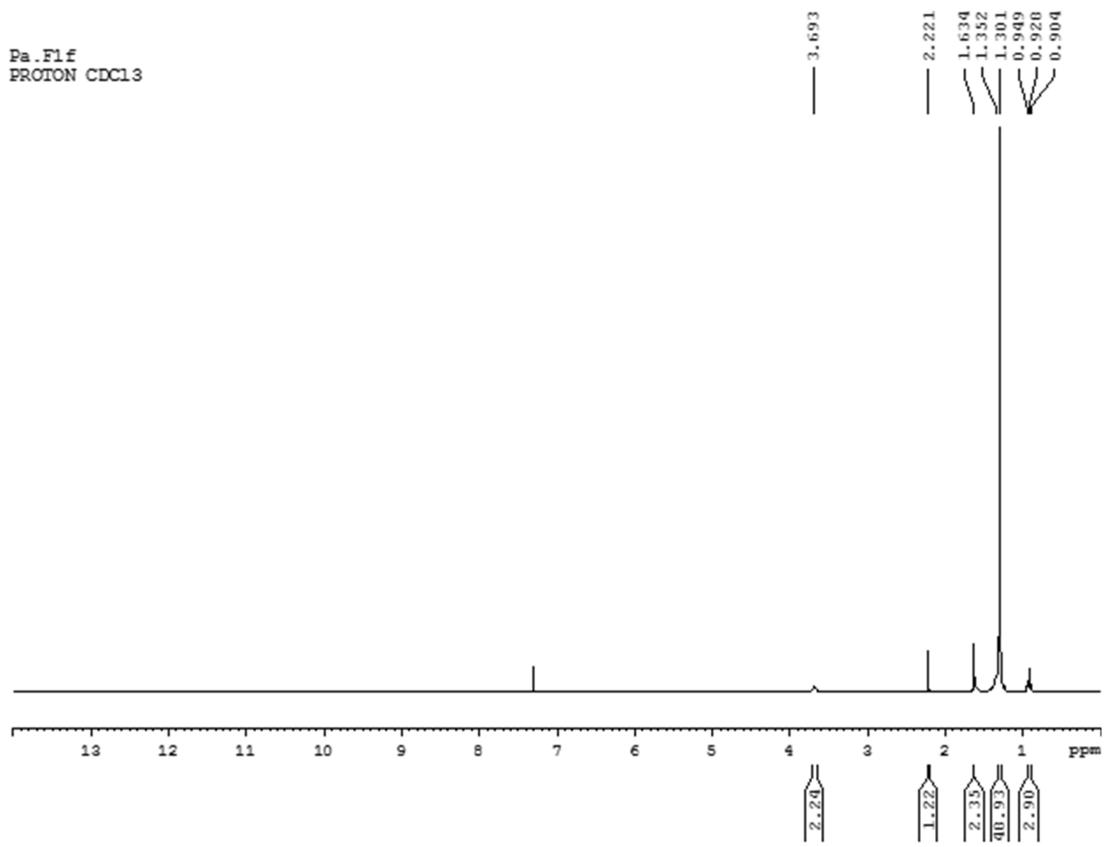

Figure S5. ^{1}H (300 MHz) NMR spectra of 1-octacosanol **16** in $CDCl_3$.

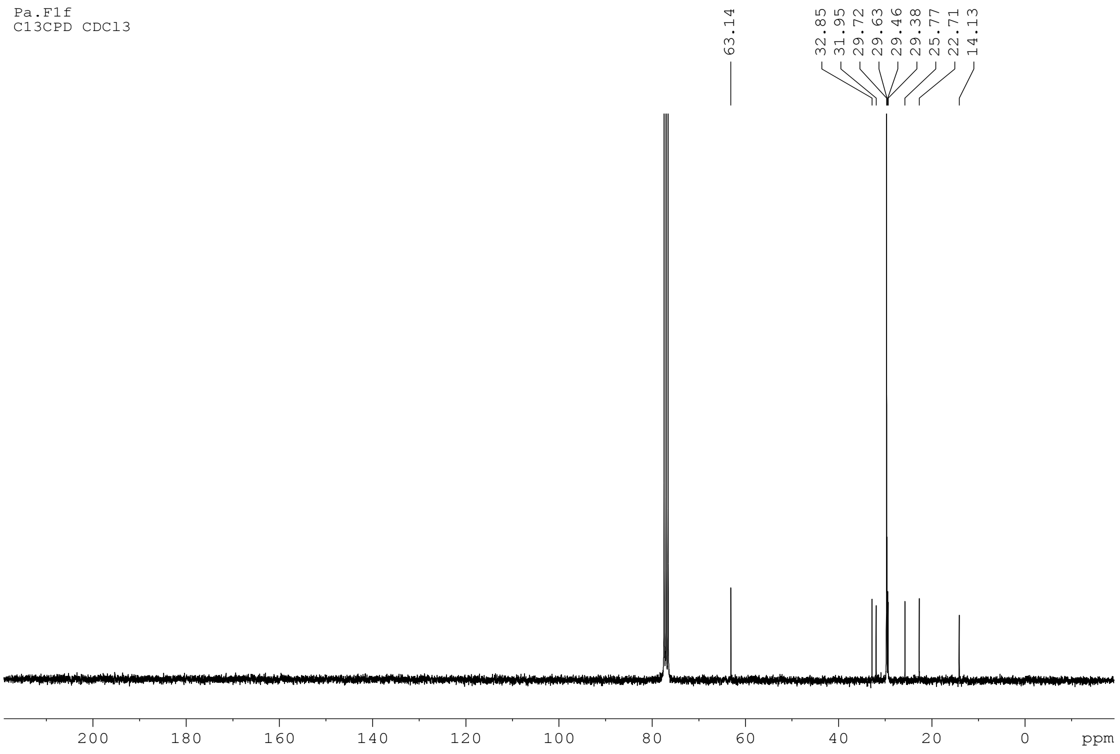

Figure S6. ^{13}C (75 MHz) NMR spectra of 1-octacosanol **16** in $CDCl_3$.
